# Therapeutic Notes

**Published:** 1897-02-13

**Authors:** 


### THERAPEUTIC NOTES.

### OZONE AS A THERAPEUTIC AGENT.

The therapeutic uses of ozone have been the subject of several communications at the July and October meetings of the French Electro-Therapeutic Society. A method of production and administration has been devised by Labbé and Oudin which appears to give satisfactory results, both as regards medicinal efficiency and comfort in inhalation. The ozone is generated in a specially designed tube by means of the silent discharge of a Rühmkorff coil. The proportion of the aërial oxygen converted into ozone in the tube appears to vary directly as the length of the spark given by the coil. The irritating effects are obviated by commencing the inhalation with the patient some distance from the tube, which he gradually approaches as tolerance is established. The authors find that ozone has a remarkable curative effect upon whooping-cough, causing the paroxysms to rapidly diminish in frequency and eventually to disappear; this result is confirmed in an independent paper by Caillé of New York. As regards tuberculosis the evidence is contradictory, Labbé and Oudin recording marked amelioration while Caillé finds the remedy ineffectual; both, however, find that it produces an increase in the oxyhæmoglobin of the blood. Labbé and Oudin claim that it also improves the body weight and the respiratory capacity. Caillé's apparatus consists of a double glass cylinder, the inner being closed and containing an aluminium plate, while the whole is surmounted by a cone of the same metal. He finds that ozone exerts a most favourable influence in cases of chlorosis and anæmia, and states that its action surpasses in such cases that of any other remedy. He further claims that its oxidising properties render it a valuable adjunct in the treatment of diphtheria and other infectious fevers.

